# Effects of cortisol administration on craving during in vivo exposure in patients with alcohol use disorder

**DOI:** 10.1038/s41398-020-01180-y

**Published:** 2021-01-05

**Authors:** Leila M. Soravia, Franz Moggi, Dominique J.-F. de Quervain

**Affiliations:** 1 Translational Research Center, University Hospital of Psychiatry, University of Bern, Bern, Switzerland; 2 Südhang Clinic, Kirchlindach, Switzerland; 3 Division of Cognitive Neuroscience, University of Basel, Basel, Switzerland

**Keywords:** Clinical pharmacology, Human behaviour, Addiction

## Abstract

Alcohol-associated memories and craving play a crucial role in the development and maintenance of alcohol use disorder (AUD). As treatment options are limited in AUD, novel treatment strategies focus on the manipulation of alcohol-associated memories. The stress hormone cortisol affects various memory processes, and first clinical studies have shown that it inhibits the retrieval of disorder-specific memories and enhances extinction memory. This study aimed to investigate the effects of a single oral administration of cortisol on craving in patients with AUD during repeated in vivo exposure to alcohol pictures and the preferred alcoholic drink. In a double-blind, block-randomized, placebo-controlled cross-over design, 46 patients with AUD were treated with two sessions of in vivo exposure to alcohol. Cortisol (20 mg) or placebo was orally administered 1 h before each test day. Craving, stress, and cortisol were repeatedly measured during exposure sessions. Results show, that cortisol administration had distinct effects on craving depending on the severity of AUD and test day. While cortisol administration significantly enhanced craving during exposure on the first test day in patients with less severe AUD, it reduced craving in patients with more severe AUD. Independent of the cortisol administration, repeated in vivo exposure reduced craving from test day 1 to test day 2. In conclusion, adding cortisol to in vivo exposure might be a promising approach for reducing the strength of alcohol-associated memories and might promote the consolidation of extinction memory in patients with severe AUD. However, the differential effect of cortisol on craving depending on AUD severity cannot be conclusively explained and highlights the need for future studies elucidating the underlying mechanism.

## Introduction

Alcohol use disorder (AUD) is a severe chronic illness with a multifactorial etiology^1^, characterized by high rates of relapse even after intensive residential treatment^2^. Despite significant progress in the development of efficacious psychological and pharmacological treatments for AUD, 1-year relapse rates remain with more than 50% very high^3^ and the prognosis regarding drinking outcome deteriorates significantly with each additional detoxification treatment^4,5^. Strong memories about cues (e.g., people, places, things, emotions) that are repeatedly associated with alcohol use can promote craving and compulsive alcohol taking and are a primary trigger of relapse^6,7^. With increasing duration and severity of AUD, these cues may also initiate the subconscious habitual and compulsive behaviors associated with obtaining and taking alcohol that further increases the likelihood of a full-blown relapse^8^. Therefore, learning and memory processes such as acquisition, consolidation, and retrieval play a crucial role in the development and maintenance of AUD. Accordingly, new treatment approaches have focused on the manipulations of learning and memory processes, including extinction and reconsolidation processes, to either strengthen or weaken the memory^6^.

In the context of exposure-based therapy, the process of extinction is of particular interest. Repeated or prolonged exposure to cues in the absence of alcohol ingestion can lead to the formation of a new “extinction” memory. Like other forms of learning, extinction acquisition is followed by a consolidation phase of the extinction memory where a cue is not associated with alcohol use anymore^6,9^. Even though there is some evidence from clinical studies that cue extinction approaches, such as exposure techniques in cognitive behavioral therapy, can reduce some of the conditioned physiological effects induced by drug cues and reduces subjective levels of craving^10,11^, many of the patients do not respond to treatment, or achieve only partial remission of symptoms^12^. Consequently, more recent research has focused on pharmacological manipulations that might be used in conjunction with extinction to help individuals to reduce relapse and maintain abstinence. Thus, drugs with the potential to enhance extinction like glucocorticoids (cortisol in humans)^13–16^ might be promising candidates to enhance exposure therapy and reduce alcohol-taking behavior.

Glucocorticoids are stress hormones released from the adrenal cortex that affect various memory processes. Despite the growing body of research on the effects of stress and glucocorticoids on memory processes, there are a number of inconsistencies indicating that such effects depend on many factors, such as the memory phase under study, time and dose of intervention, level of emotional arousal or gender^17–24^. Studies in animals and humans have shown that glucocorticoids inhibit memory retrieval while at the same time they enhance the consolidation of new memories and facilitate memory extinction processes^13,14,25–28^. Emotionally arousing information has been shown to be especially sensitive to this glucocorticoid effects^26^, which was the basis for several clinical studies investigating whether glucocorticoids can reduce the retrieval of aversive disorder-specific memories in patients with different psychiatric disorders. The acute administration of glucocorticoids reduced aversive disorder-specific memory retrieval, which was shown in reduced fear symptoms in patients with anxiety disorders^14^, reduced intrusions in chronic post-traumatic stress disorder^29^ and reduced craving in heroin-dependent patients^30^ during exposure. In addition, the conjunction of endogenously or exogenously elevated glucocorticoid levels and exposure-based therapy promoted the consolidation of fear extinction in patients with anxiety disorders^13,15,31^. Whereas the majority of randomized clinical trials have shown beneficial effects in PTSD and phobias, it has to be noted that the evidence comes from rather small proof‑of‑concept studies and there have been reports with weak or absent effects^19^.

Disorder-specific memories play a crucial role in the development and maintenance of various psychiatric disorders. In AUD, the confrontation with an alcohol-related stimulus invariably provokes the retrieval of associated alcohol-related memories that might lead to increased craving and urge to drink. Therefore, the administration of glucocorticoids could result in reduced retrieval of addiction memory and, thereby, reduce feelings of craving.

In the present randomized placebo-controlled double-blind cross-over study we examined the effects of the acute administration of glucocorticoids on craving in patients with AUD attending a cue exposure treatment session. We expect that patients receiving cortisol prior to exposure treatment will report less craving than patients receiving placebo.

## Materials and methods

### Participants

Detoxified patients with AUD attending a twelve-week abstinence-oriented residential treatment program for AUD in a specialized treatment center (Clinic Suedhang) were asked to participate in the study. Forty-eight patients with AUD according to ICD-10 gave written informed consent to participate in this randomized placebo-controlled double-blind cross-over study. A sample size of 44 patients was estimated on the basis of the assumption of a medium effect size of *d* = 0.5 at an alpha level of 0.05 and a power of 0.9. Inclusion criteria consisted of an age older than 18, abstinent from alcohol for at least 6 weeks, and attending the 12-week abstinent-oriented inpatient treatment program at the clinic Suedhang. Criteria for exclusion were severe comorbid psychiatric disorders (e.g., major depression or schizophrenia), current medical conditions excluding participation (such as acute infectious disease), recent history of systemic or topic glucocorticoid therapy, known hypersensitivity to the investigational medicinal product (IMP) (cortisol), pregnancy, breast-feeding, inability to read and understand the participant’s information, positive alcohol test according to breathalyser. Two patients had to be excluded because of the following reasons: one due to cognitive impairment, which resulted in incomplete and erroneous processing of the questionnaires, the other one due to extreme baseline values on test day 2 in craving, which might have been caused by negative events experienced between the test days. The final sample consisted of 46 patients (12 females, 34 males) with an average age was 45.3 years (SD 11.32) and an average duration of problematic alcohol consumption of 11.9 years (SD 9.97). Detailed sample description is shown in Table 1. The local ethic committee and the Swiss agency for the authorization and supervision of therapeutic products (Swissmedic, Bern, Switzerland) approved the study (Nr: 068/2014; SNCPT 207). The study was registered with ClinicalTrials.gov (Nr: NCT02196142) and monitored by the Clinical Trial Unit (CTU) of the University of Bern. The blinding was maintained throughout the study. All participants received 80 Swiss francs as a compensation for their participation.

**Table 1:** Demographic, baseline, and clinical variables of interest.

	All (*N* = 46)	Cortisol-Placebo-Group (*N* = 23)	Placebo-Cortisol-Group (*N* = 23)	*P*-values
Females/males	12/34	7/16	5/18	0.502
Oral contraceptives yes/no	3/9	2/5	1/4	0.733
Age	44.83 (11.31)	42.61 (10.79)	47.06 (11.60)	0.185
Years of probl. drinking	11.33 (9.49)	10.17 (7.65)	12.48 (11.09)	0.417
Nr. of detoxifications	2.43 (3.12)	3.00 (4.10)	1.87 (1.55)	0.223
Group: 1 detox./2+ detox.	21/25	10/13	11/12	0.767
Days of abstinence	51.09 (24.79)	53.43 (27.35)	48.74 (22.31)	0.527
AUDIT	25.33 (4.91)	25.64 (4.26)	25.04 (4.26)	0.690
AASE	63.18 (18.88)	61.59 (18.53)	64.77 (19.52)	0.582
BSCL GSI	4.66 (0.34)	0.55 (0.37)G	0.386 (0.31)	0.114
BDI-II	8.35 (5.85)	8.35 (5.85)	7.78 (4.90)	0.519
BMI	25.61 (3.85)	26.35 (4.05)	24.87 (4.05)	0.217
AUC G Placebo	16124.13 (8859.77)			
AUC G Cortisol	78239.9 (93125.07)			
OCDS T1_1	20.0 (9.14)	20.30 (7.90)	19.70 (10.41)	0.824
OCDS T2_1	8.35 (5.85)	16.13 (7.57)	13.82 (6.94)	0.268
STAI-State T1_1	35.78 (7.76)	37.13 (8.44)	34.43 (6.94)	0.243
STAI-State T2_1	23.89 (4.13)	34.48 (8.25)	31.61 (5.02)	0.162
AUQ T1	9.61 (2.71)	9.61 (2.78)	9.61 (2.71)	1.00
AUQ T2	9.04 (2.19)	8.87 (1.96)	9.22 (2.43)	0.596

Note: Group 1 Detox: Patient group with one previous detoxification; Group 2+ Detox: Patient group with two or more previous detoxifications; years of probl. drinking: years of problematic drinking; AUDIT: Alcohol Use Disorders Identification Test; OCDS: Obessive-compulsive drinking scale; T1: exposure session 1; T2: exposure session 2; BDI-II: Beck depression inventory; STAI-State: Spielberger State Anxiety Inventory; BSCL GSI: Global severity index of the Brief Symptom Check List; AASE: alcohol abstinence self-efficacy scale; BMI: body mass index; AUC: area under the curve; CAR: cortisol awakening response; AUQ: alcohol urge questionnaire.

### Procedure and measurements

**Fig. 1:**
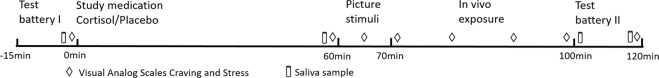
Course of study. The *x*-axis illustrates the time line of each test day. The test days were 1 week apart from each other and took place between week 6 and 8 of the abstinent-oriented inpatient treatment program for AUD. In this double-blind cross-over design, patients randomly received either cortisol or placebo at each test day (0 min). Visual analog scales for craving and stress and saliva samples were repeatedly measured. Note: Test battery I consisted of questionnaires (AASE; OCDS; AUQ), breathalyzer test, pregnancy test, heart rate monitor; Test battery II consisted of questionnaires about treatment credibility, side effects, and a debriefing.

The study took place on 2 study days (120 min duration each) between 1 pm and 4 pm at the Clinic Suedhag, Kirchlindach, Switzerland between November 2014 and June 2015. The 2 study days had the exact same procedure and were one week apart from each other (see Fig. 1). The study consisted of a pre-test assessment consisted of a pre-test screening consisting of an interview to clarify study eligibility and patients were asked to fill out several questionnaires to assess symptom severity. Test day 1 and 2 took place between week 6 and 8 of the patients’ residential treatment program and consisted of a standardized exposure procedure to neutral and alcohol pictures followed by a in vivo exposure to the preferred alcoholic drink. Each test day started with a breathalyser test to control for alcohol intake and a pregnancy test before the first saliva sample was collected and psychometric measures with the study test battery were assessed (see Fig. 1). After the oral administration of either 20 mg of hydrocortisone or placebo a resting period of 1 h followed allowing the absorption of the study medication. After the resting time period patients were presented for 10 min with neutral photographs (5 min) taken from the international affective picture system (IAPS; University of Florida, NIMH Center for the Study of Emotion and Attention, Gainsville, Fl) and alcohol photographs (5 min) on a computer. Immediately following the presentation of the neutral and alcohol photograph block, patients were asked to rate the pictures for valence, arousal, and craving using Visual Analog Scales (VAS). A standardized in vivo exposure task, consisting of the confrontation to the preferred alcoholic drink followed. The in vivo exposure task consisted of three consecutive phases: (i) looking at the preferred alcoholic drink in the bottle; (ii) opening the bottle and pouring it in a glass; (iii) holding the glass and smelling at the alcoholic drink. Again, patients were asked to rate each phase for craving and stress using VAS between the different exposure components (Fig. 1). At the end of each test day participants were interviewed regarding their current wellbeing and asked whether they think they received cortisol or placebo.

#### Study medication, randomization, and blinding

The participants were allocated randomly by the time of study entry to receive either oral cortisol (20 mg, two tablets each of 10 mg of hydrocortisone; Galepharm, Küsnacht, Switzerland) or placebo (two similar-looking tablets; Galepharm Küsnacht, Switzerland) at the first testing day. Due to the overencapsulation of the medication and the administration of just one capsule at visit 1 and one further capsule at visit 2, patients were not able to detect differences between the study medications (cross-over design). This dose of cortisol has been used in previous studies investigating the effects of a single administration of cortisol on phobic fear^14,15^ and craving in patients with heroin addiction^30^. After a washout period of 7 days, participants received on the second testing day the treatment (cortisol or placebo) that they had not received on the first testing day. The preparation of study medication and blinding was performed by the Pharmacy of the University Hospital Bern according to Good Clinical Practice (GCP). Randomization was stratified in a counterbalanced way according to order of medication (i.e., either IMP or placebo first). The IMP and the placebo were encapsulated in identically looking capsules. At testing day 1 eligible patients were allocated to the treatment group (i.e., either IMP or placebo first) following the order of the randomization list. The randomization number was listed in the CRF.

#### Saliva cortisol measurement

Four saliva samples were collected using the Salivette (Sarstedt Inc., Rommelsdorf, Germany) during each test day. A baseline saliva sample was taken immediately before substance administration, 1 h after the administration of the study drug, one after in vivo exposure and at the end of the debriefing session (see Fig. 1). After each experimental session, samples were stored at −20 °C. For biochemical analyses of free cortisol concentration, saliva samples were thawed and spun at 3000 revolutions per minute for 10 min to obtain 0.5 to 1.0 ml of clear saliva with low viscosity. Salivary cortisol concentrations were determined by a commercially available chemiluminescence immunoassay (CLIA; IBL, Hamburg, Germany). Inter- and intra-assay coefficients of variation were both below 8%.

#### Diagnostic assessment

All patients were diagnosed and screened for medical conditions at residential treatment admission from a psychiatrist in charge of the clinic Südhang. The patients’ characteristics and severity of alcohol dependence were assessed with the act-info (addiction, care, and therapy information: a nationwide documentation system for clients of Swiss drug and addiction help centers^32^, interview that includes questions on socio-demographic characteristics, such as age, gender, and employment; indices and severity of substance use and its consequences; the number of previous detoxifications; and psychological and social functioning. This interview is a nationwide instrument used during attendance and discharge of any inpatient treatment program for addictive disorders.

The Alcohol Use Disorders Identification Test (AUDIT) as part of the act-info assessment was used to assess the severity of drinking problems^33^.

Furthermore, the following questionnaires are assessed: Brief Symptom Check List (BSCL^34^); Alcohol abstinence Self-efficacy (AASE-G^35^); Beck Depression Inventory (BDI-II^36^).

#### Self-report measures during test day 1 and 2

Craving (primary outcome): Acute subjective craving as reaction to the pictures and alcohol cues were repeatedly measured using visual analog scales (VAS) ranging from 0 (lowest level) to 10 (highest level). Craving was further measured using the self-rating Alcohol Urge Questionnaire (AUQ^37^) and the Obsessive Compulsive Drinking Scale (OCDS^38^).

Stress: acute stress as reaction to the pictures and alcohol cues were repeatedly measured using visual analog scales (VAS) ranging from 0 (lowest level) to 10 (highest level).

State anxiety: state anxiety was measured before substance administration using the German version^39^ of the Spielberger State Anxiety Inventory (STAI-state)^40^, which measures subjective anxiety at the moment of assessment.

Treatment credibility and possible side effects: patients were asked after each treatment session whether they believed they were assigned to active medication or placebo. Furthermore, they were asked to report any psychological or physiological side effects of the study drug after each exposure session. None of the patients reported adverse side effects due to drug administration, nor was there any group difference in the patients’ beliefs in having received the active medication or placebo at test day 1 and 2 (*P* ≥ 0.277).

### Statistical analysis

Data were entered by blinded research assistants into SPSS version 24.0 statistical software package. Group differences in demographic and clinical characteristics, and state anxiety before exposure sessions were analyzed with unpaired *t*-tests and the visual analog scale craving with Mann–Withney U test, as they were not normally distributed. The variables of interest (VAS craving, VAS stress, OCDS, cortisol) were repeatedly measured (see Fig. 1) over each testing day and analyzed with multivariate repeated-measures ANOVA. Dependent variables were the measurements of the variables of interest and independent variables were the study medication (placebo or cortisol) and treatment order (T1 Placebo/T2 Cortisol vs. T1 Cortisol/T2 Placebo). The number of previous detoxifications is a strong predictor for relapse after residential treatment and reflects the severity of the disorder^4,5^. Thus, according to the number of previous detoxification, an additional group variable for the severity of AUD was built (1 previous detoxification (*N* = 21) compared to 2 and more previous detoxifications (*N* = 25)). Partial correlations were calculated for the associations between craving, stress, and cortisol.

The areas under the curve (AUC) were calculated with the trapezoid formula, aggregating the five measurements of the cortisol saliva samples^41^. AUC G, area under curve with respect to the ground, and AUC I, area under curve with respect to increase were calculated. Treatment credibility was analyzed with *X*^2^ tests. All tests were two-tailed and a probability of <0.05 was considered statistically significant.

## Results

### Effects of study medication on salivary cortisol levels

There was a significant main effect of study medication (cortisol/placebo) on salivary cortisol levels, with significant higher cortisol levels under cortisol treatment at the four-time points (60, 80, 100, 120 min) after substance administration compared to placebo treatment (*F*_2.3, 102.5_ = 19.09; *P* < 0.001; Supplementary Figure S1). There was no difference in the cortisol level before substance administration at each test day (*t*_45_ = 0.151; *P* = 0.881).

### Possible group effects according to the treatment order and AUD severity

The patients were randomly assigned to either receive first placebo then cortisol (T1 Placebo/T2 Cortisol: *N* = 23; 5 female) or first cortisol and at the second test day cortisol (T1 Cortisol/T2Placebo: *N* = 23; 7 female). The two groups did not differ regarding any demographic, clinical nor baseline measurements at test day 1 (Table 1). Neither did the two groups regarding AUD severity (1 previous detoxification vs. 2 and more previous detoxifications) differ regarding any demographic, clinical nor baseline measurements at test day 1, except for the number of previous detoxifications (Supplementary Table S1).

### Effects of cortisol on craving

**Fig. 2:**
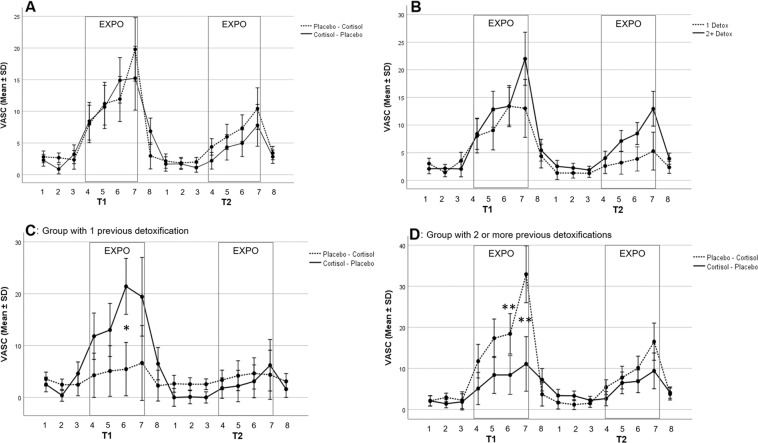
Effects of cortisol administration on craving during in vivo exposure to alcohol in patients with AUD. **A** Cortisol did not reduce craving during in vivo exposure, but repeated exposure reduced craving from T1 to T2 independent of cortisol administration. **B** Patients with a history of only one previous detoxification reported less craving during both exposure sessions compared to patients with 2 or more previous detoxifications. **C** Patients with only 1 previous detoxification receiving cortisol in the first exposure session reported significantly more craving compared to the placebo group. **D** Patients with 2 or more previous detoxification receiving cortisol in the first exposure session reported less craving compared to the placebo group. Values are depicted as mean ± SD. Note: EXPO: in vivo exposure to alcohol; T1: Exposure session 1; T2: Exposure session 2; VASC: Visual Analog Scale Craving; 1 Detox: Patient group with one previous detoxification; 2+ Detox: Patient group with two or more previous detoxifications; Placebo-Cortisol: Patient group receiving placebo at T1 and cortisol at T2; Cortisol-Placebo: Patient group receiving cortisol at T1 and placebo at T2. *P < 0.1; **P < 0.05.

For the primary outcome craving, as quantified by VAS, there was a significant interaction effect between craving, medication (Placebo vs. Cortisol), and treatment order (T1:Placebo/T2:Cortisol vs. T1:Cortisol/T2:Placebo) (*F*_2.5, 109.4_ = 6.494; *P* = 0.001) (Fig. 2A). Subsequent analysis showed that subjective craving significantly increased during in vivo exposure on test day 1 and 2 (*F*_1.5, 109.4_ = 14.497; *P* < 0.001). Independent of the medication and treatment order, there was a significant reduction in subjective craving from test day 1 to test day 2 (*F*_1, 44_ = 11.01; *P* = 0.002). However, there was no significant effect of medication (*F*_1.0, 44.0_ = 0.17; *P* = 0.681) nor treatment order (*F*_1.0, 44.0_ = 0.12; *P* = 0.730) on subjective craving (see Fig. 2A).

Subsequent repeated-measures ANOVA with the additional group variable of AUD severity showed a significant interaction effect between craving, medication (Placebo vs. Cortisol), treatment order (T1:Placebo/T2:Cortisol vs. T1:Cortisol/T2:Placebo), and AUD severity (1 previous detoxification vs. 2+ previous detoxifications) (*F*_2.5, 107.1_ = 3.90; *P* = 0.015). Subsequent analysis for the AUD severity groups at test day 1 and 2, showed that patients with only one previous detoxification receiving cortisol in the first exposure session showed an increase in subjective craving during exposure (*F*_2.3, 38.0_ = 3.23; *P* = 0.044; T1 VAS 6: *U* = 31.5, *p* = 0.093; All other VAS craving at T1 and T2: *p* > 0.05) (Fig. 2C), while the opposite effect was shown in patients with two or more previous detoxifications receiving cortisol, showing a less craving (*F*_1.7, 38.0_ = 3.25; *P* = 0.058; T1 VAS 6: *U* = 42.5, *p* = 0.048; T1 VAS 7: *U* = 41.0, *p* = 0.042; All other VAS craving at T1 and T2: *p* > 0.05) (Fig. 2D). There was no effect of substance administration on craving during the second test day in both groups (all *P* > 0.344). Independent of the time of cortisol administration, both groups showed a reduction in subjective craving from the first to the second test day (Group 1 detox: *F*_1, 20_ = 4.470; *P* = 0.047; Group 2+detox: *F*_1, 24_ = 7.930; *P* = 0.010).

### Effects of cortisol on stress

The results of the two factorial analysis of variance with repeated measurements for the visual analog scale stress, as quantified by VAS stress showed a significant effect on stress over the course of the two test days (*F*_2.6, 112.1_ = 7.63; *P* < 0.001), a significant interaction between medication and treatment order (*F*_1.0, 43.0_ = 14.42; *P* < 0.001) and an interaction between stress, medication and treatment order (*F*_2.6, 110.49_ = 2.89; *P* = 0.046) (Figure S2). Subsequent analysis showed that subjective stress changed over the course of the experiment at test day 1 and 2 (all *p* < 0.001) with a significant reduction from test day 1 to test day 2 (*F*_1.0, 45.0_ = 13.3; *P* = 0.001). Subsequent paired T-tests showed a significant reduction in subjective stress from the first to the second test day, but only during the in vivo exposure to alcohol pictures and the preferred alcoholic drink (all *p* < 0.005). Again, there was no effect of the substance administration (*F*_1.0, 44.0_ = 0.30; *P* = 0.586) nor treatment order (*F*_2.3, 101.5_ = 0.61; *P* = 0.569) on subjective stress.

### Association between craving, stress, and cortisol

Subjective changes in craving (delta craving) and stress (delta stress) within the exposure session was positively correlated with the change in cortisol levels (AUC G cortisol) only during the session when patients received placebo (Craving: Placebo condition: AUC G: *r* = 0.343; *P* = 0.019; Cortisol condition: AUC G: *r* = −0.070; *P* = 0.642; Stress: Placebo condition: AUC G: *r* = 0.518; *P* < 0.001; Cortisol condition: AUC G: *r* = −0.019; *P* = 0.901). Additional stepwise regression analysis revealed that only stress was a significant predictor for the endogenous cortisol secretion (AUC G during Placebo condition) while craving, treatment order, and severity of AUD were not significantly associated (Table 2).

**Table 2:** Stepwise regression with cortisol as the dependent variable, and cortisol, stress, treatment order, and severity of AUD as predictors of interest.

	*R²*	Corrected *R²*	∆*R²*	∆*F*	df	∆*P*	Standardized *ß*	*t*	*P*
Stress Δ	0.268	0.251	0.268	15.74	1;43	0.000	0.518	3.97	0.000

There was a significant reduction in obsessive-compulsive drinking scale (OCDS) between the first and the second exposure session (*F*_1.0, 44_ = 19.41; *P* < 0.001), which was independent of the time when the patients received cortisol or placebo (*F*_1.0, 44_ = 0.55; *P* = 0.461).

## Discussion

This study aimed to investigate the effects of a single oral administration of cortisol on craving in patients with AUD during repeated in vivo exposure to alcohol.

Cortisol administration had distinct effects on craving depending on the severity of AUD and test day. While cortisol significantly enhanced craving during exposure on the first test day in patients with only one previous detoxification, it reduced craving in patients with two or more previous detoxifications. Findings from animal and human studies point out the crucial role of memory processes for the development and maintenance of addiction^11,42^. In AUD, the retrieval of alcohol-associated memory triggers craving^6,43,44^. Thus, the severity of AUD is often reflected by the level of craving and associated relapses^5,45,46^. During exposure to alcohol cues, patients with more than two previous detoxifications reported more craving that might result from more or stronger alcohol-associated memories that are readily retrieved and elicit craving. In these patients, the administration of cortisol might have inhibited the retrieval of alcohol-associated memories during in vivo exposure and thus reduced craving. On the other side, we found the opposite effect of cortisol administration on craving in patients with only one previous detoxification. Even though there is extensive evidence that stress increases alcohol craving and the vulnerability for relapse^47–51^, findings from animal and human studies are heterogeneous regarding the involvement of cortisol in mediating these stress effects^52–54^. Another explanation for the differential effects may come from the adaptation in the hypothalamic–pituitary–adrenal (HPA) axis function through chronic alcohol use and AUD^47,55–58^. Thus, it might be possible that changes in the HPA axis depend on the severity of AUD and therefore lead to differential responses to external glucocorticoids. However, this would have to be tested with HPA-reactivity tests, such as the dexamethasone suppression test, in these patient groups. However, the distinct effect of cortisol on craving cannot be conclusively explained. Thus, more studies are needed to elucidate the underlying mechanism and the usage of cortisol as an add-on treatment for AUD needs to be further investigated with regard to AUD severity.

Independent of substance administration, repeated in vivo exposure reduced craving and stress from test day 1 to test day 2. These findings have several implications. First, the significant increase of craving and stress following in vivo exposure evidenced that after repeated alcohol administration, cues associated with the consumption of alcohol (such as the sight or smell of alcohol) can elicit conditioned responses (for reviews, see refs. ^59,60^). Furthermore, the intensity of the response is positively related to the subject’s degree of dependence^61^. This underlines that with ongoing alcohol use the associated memories become stronger and particularly difficult to disrupt. Second, exposure to such cues in the absence of alcohol ingestion, can lead to the formation of a new “extinction” memory, which is supported by our findings that only one standardized in vivo exposure sessions significantly reduced craving and stress, which in turn might reduce the probability of relapse to alcohol drinking^59,62^.

In vivo exposure to the preferred alcoholic drink significantly increased craving and stress but not salivary cortisol during both exposure sessions in the placebo condition. Chronic alcohol use and AUD is associated with adaptations in stress-related brain pathways and the HPA-axis function as well as in the autonomic arousal and reward (mesolimbic dopamine) pathways^47,55–58^. Acute alcohol administration has been shown to enhance levels of HPA-axis hormones (e.g., cortisol) in humans and animal models (for review, see ref. ^63^). As dependence on alcohol develops, HPA-axis activity appears to become dysregulated, and ongoing chronic exposure to alcohol may lead to a reduction in the responsiveness of the HPA axis to external stimuli^64,65^. While the acute and chronic alcohol intoxication and withdrawal robustly increase cortisol secretion^66,67^, a suppression of the HPA axis responsiveness with low basal levels of cortisol^68^ and a blunted cortisol responses to social stress tasks^69^ and alcohol cue exposure^70,71^ is reported in early abstinent patients with AUD. Higher cortisol response to stress appears to have protective effects and enables individuals to reduce or regulate negative affect^14,72^. This imbalance between the psychological and physiological stress response is in line with our findings, as patients reported a significant increase in craving and stress during exposure to alcohol while they showed a blunted cortisol response, which might reflect the potential impairment of the person’s ability to adequately cope with relapse-inducing stressors. However, the findings on cortisol reactivity to alcohol cue exposure are heterogeneous^69,73^, suggesting a dysfunctional HPA system rather than specific cortisol hypo-responsivity in AUD^74^, depending on the different stages of AUD such as heavy drinking, withdrawal, early abstinence, prolonged abstinence, and relapse^71^. As HPA response to stress significantly influences and modulates the affective and behavioral regulation, the alteration of the HPA-axis might have a potential impact in the vulnerability to relapse in patients with AUD.

There are some limitations, that need to be addressed. The effects of stress and cortisol on memory process depend on various factors (e.g., cortisol reactivity, gender, population, timing) highlighting the importance of well-elaborated methodology^18,23,24,75^. As cortisol has distinct effects on memory retrieval and consolidation, careful study designs are warranted. The cross-over design, complicated the analysis and led to a loss of power due to the strong habituation effect in craving from the first to the second test day. Thus, for the investigation of the acute effect of cortisol in craving only short exposures to alcohol cues would have been a better approach. Timing of glucocorticoid administration seems to be a key determinant of its effects on memory processes^18^. Thus, timing and dosage of glucocorticoid administration was based on our previous clinical studies^14,15,29,30^. However, as stated before there is evidence that patients with AUD show altered HPA-activity and often altered markers of liver function or liver disease according to the stage of AUD (acute or chronic alcohol intoxication, withdrawal, early abstinence, long-term abstinence). Thus, timing and dosage of glucocorticoid administration may need to be adjusted in patients with severe AUD and have to be tested in further studies. To evaluate possible beneficial effects on the promotion of cortisol administration on extinction memory, repeated cortisol administration in combination with exposure therapy and follow-up assessment should be planned.

Summarized, this is the first study to examine the acute effects of cortisol on craving during in vivo exposure in patients with AUD. Cortisol administration had distinct effects on craving depending on the severity of AUD, which highlights the need for future research investigating HPA-axis functioning in patients with AUD more thoroughly. Cortisol reduced craving in patients with severe AUD and repeated exposure reduced craving. Thus, adding cortisol to in vivo exposure might be a promising approach for reducing the strength of drug-associated memories and promote the consolidation of extinction memory in patients with severe AUD. However, the presented differential effect of cortisol administration on craving depending on the severity of AUD is not fully understood and highlights the need for further studies to elucidate the underlying mechanism. The findings underline the importance of the development of new treatment approaches that address neurobiological changes to learning and memory systems, which play a crucial role in the development and maintenance of addiction.

## Supplementary information

Figure S1

Table S1

CONSORT-Flow Diagram
