# Supplementary figures and images for: Effects of cortisol administration on craving during in vivo exposure in patients with alcohol use disorder
Source: Transl Psychiatry. 2021 Jan 5;11:6. doi: 10.1038/s41398-020-01180-y (PMC7791020; doi:10.1038/s41398-020-01180-y)

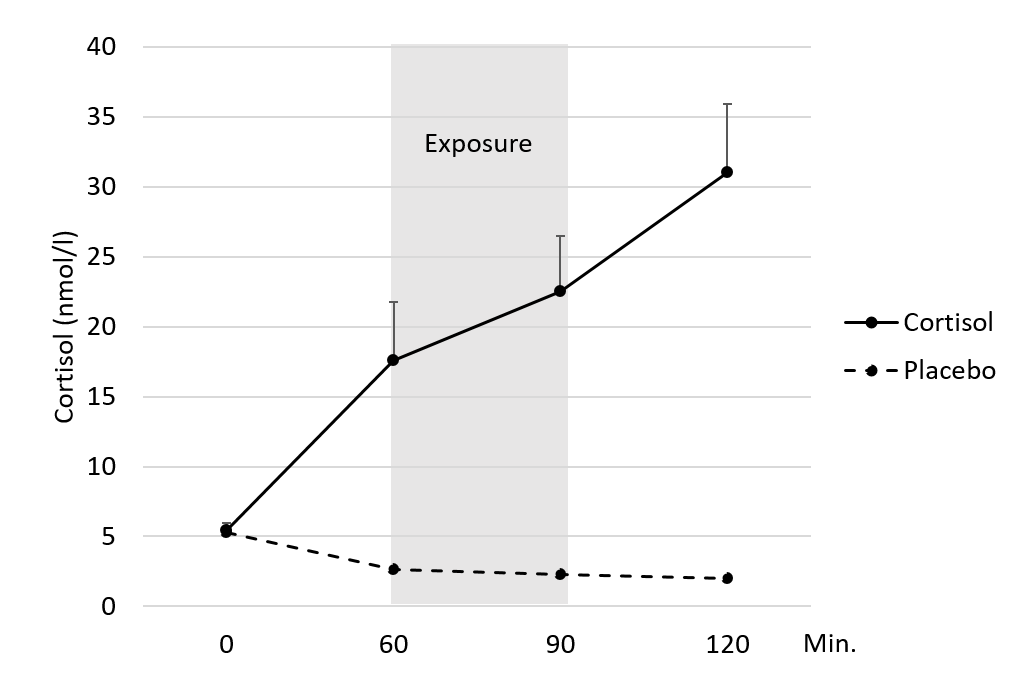

Supplement: Supplementary file 1 — Figure S1 [file 41398_2020_1180_MOESM1_ESM.tif]
