# Supplementary material for: Effects of cortisol administration on craving during in vivo exposure in patients with alcohol use disorder
Source: Transl Psychiatry. 2021 Jan 5;11:6. doi: 10.1038/s41398-020-01180-y (PMC7791020; doi:10.1038/s41398-020-01180-y)
Supplement: Supplementary file 2 — Table S1 [file 41398_2020_1180_MOESM2_ESM.docx]

**Supplements**

Table S1: Demographic, baseline and clinical variables of interest

|  | 1 detox.-Group (N = 21) | 2+ detox.-Group (N = 25) | P-values |
| --- | --- | --- | --- |
| Females/males | 7/14 | 5/20 | .305 |
| Oral contraceptives yes/no | 0/7 | 1/4 | .051 |
| Age | 43.67 (12.13) | 45.81 (10.488) | .528 |
| Years of probl. drinking | 9.33 (7.95) | 13.00 (11.09) | .195 |
| Nr. of detoxifications | 1.00 (0.00) | 3.76 (3.77) | .001 |
| Group: Cortisol-Placebo / Placebo-Cortisol | 10/11 | 13/12 | .767 |
| Days of abstinence | 52.57 (22.82) | 49.84 (26.74) | .741 |
| AUDIT | 24.66 (5.14) | 25.92 (4.72) | .400 |
| AASE | 61.50 (20.29) | 62.92 (18.06) | .920 |
| BSCL GSI | 0.45 (0.34) | 0.48 (0.35) | .764 |
| BDI-II | 8.24 (5.97) | 8.44 (5.87) | .909 |
| BMI | 25.62 (4.60) | 25.6 (3.14) | .986 |
| AUC G Placebo | 13373.61 (6293.76) | 18434, 56 (10098.98) | .053 |
| AUC G Cortisol | 99622.16 (108776.01) | 60278.86 (75261.19) | .170 |
| OCDS T1_1 | 18.23 (9.65) | 21.48 (8.61) | .235 |
| OCDS T2_1 | 14.67 (8.16) | 15.24 (6.60) | .793 |
| STAI-State T1_1 | 34.95 (7.01) | 36.48 (8.42) | .512 |
| STAI-State T2_1 | 30.95 (4.64) | 34.80 (8.04) | .059 |
| AUQ T1 | 10.20 (3.20) | 9.12 (2.17) | .185 |
| AUQ T2 | 8.86 (2.13) | 9.20 (2.27) | .603 |

Note: Group 1 Detox : Patient group with one previous detoxification ; Group 2+ Detox : Patient group with two or more previous detoxifications; Years of probl. drinking: years of problematic drinking; AUDIT: Alcohol Use Disorders Identification Test; OCDS: Obessive-compulsive drinking scale; T1: Exposure session 1; T2: Exposure session 2; BDI-II: Beck depression inventory; STAI-State: Spielberger State Anxiety Inventory; BSCL GSI: Global severity index of the Brief Symptom Check List; AASE: Alcohol abstinence self-efficacy scale; BMI; body mass index; AUC: area under the curve; CAR: cortisol awakening response; AUQ: Alcohol Urge questionnaire
