# Supplementary material for: Effects of cortisol administration on craving during in vivo exposure in patients with alcohol use disorder
Source: Transl Psychiatry. 2021 Jan 5;11:6. doi: 10.1038/s41398-020-01180-y (PMC7791020; doi:10.1038/s41398-020-01180-y)
Supplement: Supplementary file 3 — CONSORT-Flow Diagram [file 41398_2020_1180_MOESM3_ESM.doc]

**
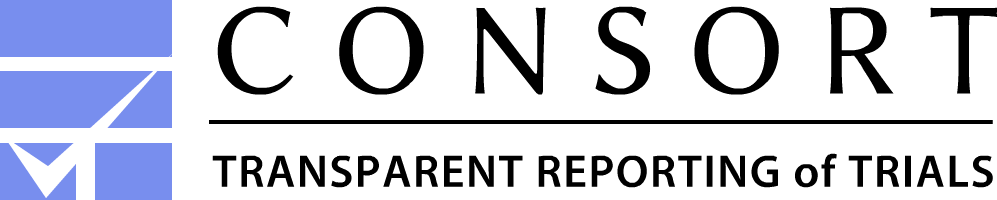
**

**CONSORT 2010 Flow Diagram[[1]](#footnote-2)**

**Analysis**

**Enrollment**

**Allocation**

Allocated to intervention A (n= 24)

**Analysis**

♦ Received allocated intervention (n= 24)

♦ Did not receive allocated intervention (give reasons) (n= 0)

Allocated to intervention B (n= 24)

♦ Received allocated intervention (n= 24)

♦ Did not receive allocated intervention (give reasons) (n= 0)

**Male:** Allocated to intervention A (n= 16)

 Received allocated intervention (n= 16)

 Did not receive allocated intervention (give reasons) (n= 0)

**Female:** Allocated to intervention (n= 8)

♦ Received allocated intervention (n= 8)

♦ Did not receive allocated intervention (give reasons) (n= 0)

**Male:** Allocated to intervention B (n= 19)

 Received allocated intervention (n= 19)

 Did not receive allocated intervention (give reasons) (n= 0)

**Female:** Allocated to intervention B (n= 5)

♦ Received allocated intervention (n= 5)

♦ Did not receive allocated intervention (give reasons) (n= 0)

Analysed (n= 23)
♦ Excluded from analysis (crisis between the two test-days, extreme baseline values ​​on test day 2 in craving) (n= 1)

Analysed (n= 23)
♦ Excluded from analysis (cognitive impairment, incomplete and erroneous processing of the questionnaires) (n= 1)

**Male:** Analysed (n= 16)
♦ Excluded from analysis (give reasons) (n= 0)

**Female:** Analysed (n= 7)
♦ Excluded from analysis (give reasons) (n= 1)

**Male:** Analysed (n= 18)
♦ Excluded from analysis (give reasons) (n= 1)

**Female:** Analysed (n= 5)
♦ Excluded from analysis (give reasons) (n= 0)

Assessed for eligibility (n= 48)

Excluded (n= 0)

♦  Not meeting inclusion criteria (n= 0)

♦  Declined to participate (n= 0)

♦  Other reasons (n= 0)

48

Analysed (n= )
♦ Excluded from analysis (give reasons) (n= )

Analysed (n= )
♦ Excluded from analysis (give reasons) (n= )

Analysed (n= )
♦ Excluded from analysis (give reasons) (n= )

Analysed (n= )
♦ Excluded from analysis (give reasons) (n= )

Analysed (n= )
♦ Excluded from analysis (give reasons) (n= )

Analysed (n= )
♦ Excluded from analysis (give reasons) (n= )

**Analysis**

1. Sex used as an example (male/female) but other PROGRESS characteristics could be used [↑](#footnote-ref-2)
